# Lithiophilic Silver Coating on Lithium Metal Surface for Inhibiting Lithium Dendrites

**DOI:** 10.3389/fchem.2020.00109

**Published:** 2020-02-21

**Authors:** Zefu Zuo, Libin Zhuang, Jinzhuo Xu, Yumeng Shi, Chenliang Su, Peichao Lian, Bingbing Tian

**Affiliations:** ^1^The Higher Educational Key Laboratory for Phosphorus Chemical Engineering of Yunnan Province, Faculty of Chemical Engineering, Kunming University of Science and Technology, Kunming, China; ^2^International Collaborative Laboratory of 2D Materials for Optoelectronics Science and Technology of Ministry of Education, Institute of Microscale Optoelectronics, Shenzhen University, Shenzhen, China

**Keywords:** Li metal batteries, thermal evaporation, Li dendrites, Ag layer, lithiophilic layer

## Abstract

Li metal batteries (LMBs) are known as the ideal energy storage candidates for the future rechargeable batteries due to the high energy density. However, uncontrolled Li dendrites growing during charge/discharge process causes extremely low coulombic efficiency and short lifespan. In this work, a thin lithiophilic layer of Ag was coated on the bare Li surface via a thermal evaporation method, which alleviated volume variations and suppressed Li dendrites growth during cycling. As a result, a long lifespan of 250 h at a current density of 1 mA cm^−2^ was achieved in the symmetric cell when using the Ag-modified Li foil (Ag@Li). The LiFePO_4_|Li full cell demonstrated an excellent cycling performance with a high specific capacity of 131 mAh g^−1^ even after 300 cycles at 0.5 C. This study offers a suitable method for stabilizing Li metal anodes in LMBs.

## Introduction

The advent of electric vehicles (EVs) has spurred the need for high energy density batteries (Tarascon and Armand, 2011; Xiang et al., 2011; Li et al., 2016; Xue et al., 2019a). The capacity of the widely used lithium ion batteries (LIBs) using traditional graphite anodes with 372 mAh g^−1^ are difficult to catch up with the demand of the current energy market (Whittingham, 1976; Choi et al., 2012; Goodenough and Park, 2013; Choi and Aurbach, 2016; Schmuch et al., 2018). Li metal is recognized as one of perfect anodes for LIBs owing to its lowest reduction potential (−3.04 V vs. the standard hydrogen potential electrode) and ultrahigh theoretical specific capacity (3,860 mAh g^−1^) (Xu et al., 2014; Yang et al., 2015; Lin et al., 2017; Wang A. et al., 2018; Xue et al., 2019b). Nevertheless, working risks come from the uncontrolled Li dendrites formation and side-reactions of highly active Li with the electrolyte, which will induce Li metal batteries (LMBs) short circuit (Aurbach et al., 2002; Qian et al., 2015; Takeda et al., 2016; Yan et al., 2016; Guo et al., 2017; Wang H. et al., 2017; Sahalie et al., 2019). To settle these problems, great efforts have been devoted to improve the stability of Li metal anode. For instance, diverse electrolyte additives were used to prevent side reactions of Li with organic electrolytes (Grande et al., 2015; Li et al., 2015; Zheng et al., 2017; Zhang X. Q. et al., 2018). Solid-state electrolytes with high mechanical strength and lithiophilic three-dimensional (3D) hosts with increased specific surface area were also developed for LMBs to enhance the safety of Li anodes (Han et al., 2017; Wang S. H. et al., 2017; Jiang et al., 2018; Qi et al., 2018; Yue et al., 2018; Chen X. et al., 2019). Although certain improvements in stability of Li metal anodes have been made, some shortcomings should be considered when they are applied in practical application of LMBs. In simple terms, large voltage hysteresis appeared in the batteries using solid electrolyte due to the poor ionic conductivity of solid electrolyte and the large interfacial resistance of the electrode and solid electrolyte (Kamaya et al., 2011; Bouchet et al., 2013). Electrolyte additives for SEI stabilization can only reinforce SEI formation and prevent dendrite propagation (Cheng et al., 2017; Zhang X. Q. et al., 2018). 3D skeletal structures were prone to collapse under high current densities (Ye et al., 2017; Wang L. et al., 2018). Expect from the above methods, covering the surface of the Li foil with a lithiophilic layer is another effective way to protect the Li metal anode. Till now, it has been reported that ZnO/carbon nanotube (CNT), Au, S, P, Sn, and Si have been reported to inhibit Li dendrites (Lin et al., 2018; Zhang X. Q. et al., 2018; Guo et al., 2019; Liang et al., 2019; Xia et al., 2019). Chen et al. used sulfur vapor to react with Li then transform into Li_2_S on the Li surface (Chen H. et al., 2019). The Li_2_S@Li|Li_2_S@Li cell maintained an overvoltage of 95 mV after 600 h at the current density of 2 mA cm^−2^ and fixed capacity of 5 mAh cm^−2^. The high ionic conductivity of Li_2_S relieved non-uniform Li ions flux and suppressed Li dendrites, but it is almost an insulator. Kim et al. proposed a method that phosphorene is added dropwise on the Li foil surface (Kim et al., 2018). The phosphorene layer located above the Li surface spontaneously reacts with Li to form Li_3_P. The phosphorene-coated Li metal electrode displayed a constant capacity of 1,000 mAh g^−1^ with no capacity reduction even after 50 cycles for the Li-O_2_ battery. The Li_3_P protective layer effectively suppressed Li dendrites in terms of thermodynamics. However, the Li_3_P artificial solid electrolyte interphase (SEI) layer constructed by a simple spin coating method is not strong enough to continually resist the growth of Li dendrites. Tang et al. deposited Si on the Li surface by using a magnetron sputtering system. The bottom of the Li foil was heated to make Si reacting with Li and a Li_x_Si layer was obtained (Tang et al., 2018). In the symmetric cells, stable cycling performance was achieved for the Li_x_Si@Li electrode over 400 h at the current density of 1 mA cm^−2^. It was demonstrated that the coated Li_x_Si layer led Li ions distributed uniformly on the surface of the Li metal anode during cycling. Nevertheless, the fabrication technology of the Li_x_Si@Li electrode is quite complicated, which hinders its practical application. Generally, metals have excellent electrical conductivity. It is well-known that Ag has a good wettability with Li and excellent electrical conductivity compared to other metals in the previous report (Zhang X. Q. et al., 2018; Song et al., 2019).

Herein, a thin Ag layer was coated on the Li foil surface (Ag@Li) by thermal evaporation. Due to this modified Ag layer, Li ions deposited uniformly on the surface and therefore Li dendrites were effectively suppressed during cycling. The symmetric cells using the Ag@Li electrodes displayed superior cycling performance for 85 h at a high current density of 5 mA cm^−2^. The full cells assembled with LiFePO_4_ (LFP) as cathodes exhibited a capacity retention of 92% after 300 cycles.

## Experimental Section

### Preparation of the Ag@Li Electrode

A 0.6 mm-thick Li tape (Tianjin Zhongneng Lithium Co. Ltd.) was cut into Li discs with a diameter of 16 mm in an argon-filled glovebox. The cut Li discs were transferred to the thermal evaporation equipment connected with the glovebox. The Ag@Li discs were fabricated by using a ZD-450 single-chamber six-source resistive evaporation equipment with Ag metal as source at a current density of 88 mA under 1.0 × 10^−4^ Pa. Specifically, the Li discs were fixed on the substrate holder, and the Ag metal was placed in the tungsten boat. Deposition rate set to 0.1 Å s^−1^.

### Materials Characterization

The microstructure and element distribution of the bare Li and the Ag@Li discs before and after electrochemical plating, and after 50 cycles was observed by scanning electron microscope (SEM, TESCAN MIR A3) equipped with energy dispersive spectroscopy (EDS). The X-ray diffraction (XRD) analysis was characterized using a Rigaku Ultima IV at 40 kV and 40 mA with a scan rate of 5^o^ min^−1^ (Cu Kα X-ray radiation source).

### Electrochemical Characterization

CR2016-type coin cells were assembled using Ag@Li discs as the working electrodes and bare Li foil as the counter electrodes. 1 M lithium bis(trifluoreomethane)sulfonamide (LiTFSI) in 1,3-dioxolane/1.2-dimethoxyethane (DOL/DME, 1:1 by volume) with 1% LiNO_3_ and 1 M LiPF_6_ in ethylene carbonate/diethyl carbonate (EC/DEC/EMC, 1:1:1 by volume) were used as the electrolytes. The amount of electrolyte was 50 μL. Electrochemical performance of the Li|Li symmetric cells and LiFePO_4_|Li full cells were tested using Shenzhen Neware battery testing system. Electrochemical impendence spectroscopy (EIS) measurements were investigated by IVIUMnSTAT electrochemical workstation with a frequency range from 100 kHz to 0.01 Hz at voltage amplitude of 10 mV. The active material LiFePO_4_, super P conductive carbon and polyvinylidene fluoride (PVDF) were mixed in the weight ratio of 8:1:1 as the cathode materials. The loading mass of LiFePO_4_ was ~1 mg cm^−2^. Galvanostatic charge/discharge cycling tests of the full cells were carried out in a potential range of 2.4–4.2 V at 0.5 C. The rate performance of LiFePO_4_|Li full cell was tested under 0.5, 1, 2, 3, 5, 10 C then back to 1 C.

## Results and Discussion

**Figure 1:**
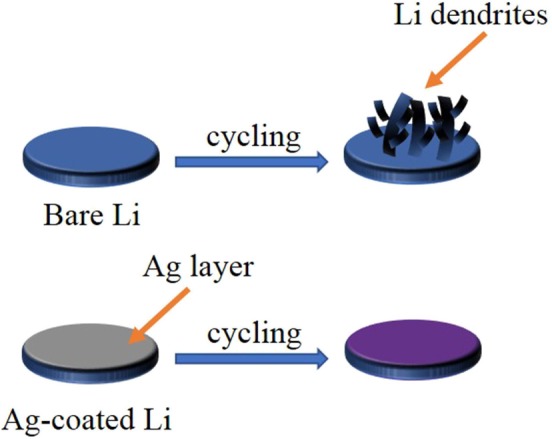
Schematic illustration of bare Li and Ag@Li discs during cycling process.

Figure 1 shows the schematic illustration of the bare Li and the Ag@Li electrodes during cycling process. For the unprotected Li disc, an uneven SEI layer appeared due to the inhomogeneous Li ions flux, resulting in Li dendrites growth, which leads to the internal short circuit of the batteries. In contrast, with the deposition of Ag on Li metal (Ag@Li), the electrode surface is much flatter during the charge/discharge process. The lithiophilic Ag layer can induce uniform deposition of Li ions, therefore inhibiting the growth of Li dendrites and achieving excellent cycling performances of LMBs.

**Figure 2:**
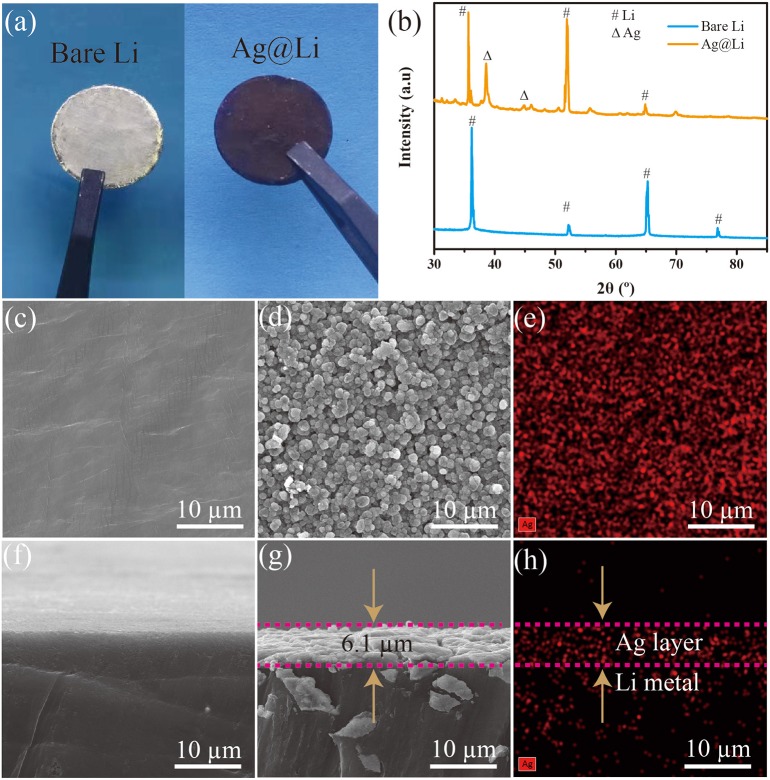
**(a)** Optical photographs and **(b)** XRD patterns of bare Li and Ag@Li electrodes; **(c)** SEM images of the surface and **(f)** cross section of bare Li electrode; **(d)** SEM images of the surface and **(g)** cross section of the Ag@Li electrode; **(e)** Element distributions of Ag on the surface and **(h)** cross section of Ag@Li electrode.

As illustrated in Figure 2a, there is a distinct change in color from silvery white to black after Ag layer coated on the Li foil. From the XRD results in Figure 2b, the diffraction peaks at 38.1° and 44.3° refer to the (111) and (220) crystal planes of Ag, indicating that Ag layer have been coated on the bare Li disc. SEM images were characterized to observe the microstructure of the bare Li and the Ag@Li anodes. From Figures 2c,f, the surface of bare Li is very smooth without impurities. After the thermal evaporation deposition of Ag, the Li surface is covered with Ag particles with a size of about 1 μm (Figure 2d). As shown in Figure 2g, a thin Ag layer with the thickness of around 6.1 μm is coated uniformly on the Li metal surface. Ag element distributions shown in Figures 2e,h illustrate that the Ag particles and the coated Ag layer are also distributed uniformly on the Li surface, corresponding with the SEM images in Figures 2d,g.

**Figure 3:**
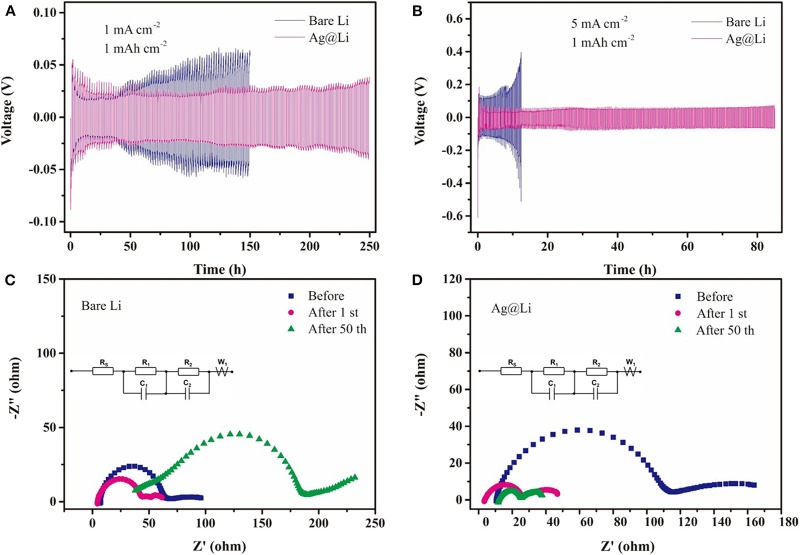
Voltage-time profiles of the Li plating/stripping process in symmetric Li|Li and Ag@Li|Ag@Li cells at different current densities of **(A)** 1 mA cm^−2^ and **(B)** 5 mA cm^−2^. The capacity is fixed at 1 mAh cm^−2^. EIS curves and equivalent circuit of the symmetric cells with **(C)** bare Li and **(D)** Ag@Li electrodes during cycling at the current density of 1 mA cm^−2^ and a fixed capacity of 1 mAh cm^−2^.

The symmetric cells are constructed to test the variation of polarization voltage using the bare Li and the Ag@Li electrodes. As shown in the Figure 3A, the Li|Li symmetric cell appears an increased overpotential after 50 h. By comparison, the cell using the Ag@Li electrode exhibits larger polarization overpotential during the initial several cycles. However, the voltage hysteresis of the symmetric cell with the Ag@Li electrode decreases in the first 50 h and keeps stable at 38 mV even after 250 h, indicating that the cell with the Ag@Li electrode possessed good cycling electrochemical performance. When it comes to higher current density (5 mA cm^−2^) (Figure 3B), the cell with the bare Li anode shows considerable overpotential (from 140 to 400 mV) during Li plating/string process. In contrast, the Ag@Li electrode exhibits much lower overpotential and more stable electrochemical performance. It is worth noting that the cell with the bare Li electrode gets short-circuited in only 12 h at high current density of 5 mA cm^−2^ due to the Li dendrite growth. As for the Ag@Li electrode, the Ag layer constructed on the surface of Li metal leads to uniform deposition of Li ions, which is effectively improve the stability of Li metal. EIS curves and equivalent circuit of the symmetric cells with bare Li and Ag@Li electrodes during cycling at the current density of 1 mA cm^−2^ are shown in Figures 3C,D. The electrolyte resistance (Rs) of the Li|Li cell decrease first and then increases to 38 Ω, while the Rs of Ag@Li|Ag@Li cell is smaller than that of the Li|Li cell after 50 cycles, respectively. The SEI resistance (R_f_) is consisted of the high frequency region R_1_ and the low frequency region R_2_. The R_f_ of the Li|Li cell shows a increasing trend and finally comes to as high as 154 Ω after 50 cycles. As for the Ag@Li|Ag@Li cell, R_f_ is nearly 120 Ω before cycling but it decreases sharply to 33 Ω and keep steadily after 50 cycles.

**Figure 4:**
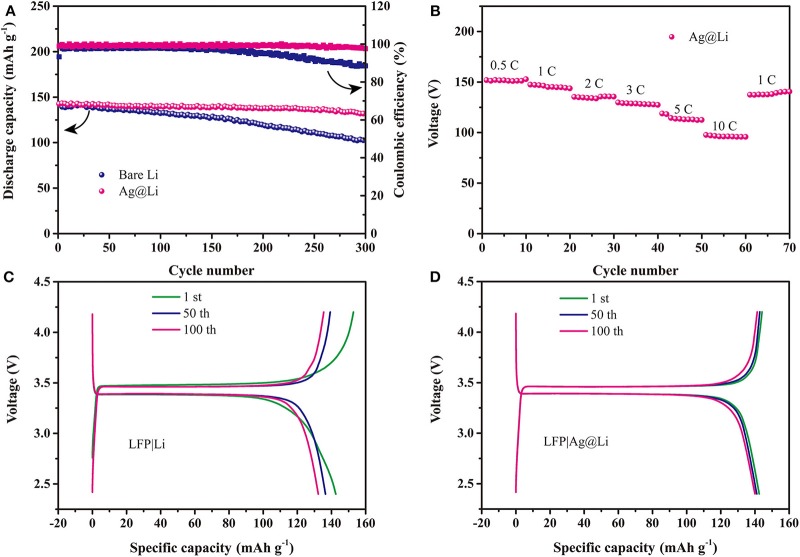
**(A)** Cycling performance of the LFP|Li and LFP|Ag@Li cells at 0.5 C. **(B)** Rate performance of the LFP|Ag@Li cell. Charge/discharge profiles of the **(C)** LFP|Li and **(D)** LFP|Ag@Li cells after 1st, 50th, and 100th cycles at 0.5 C.

Figure 4A displays the cycling performance of the LFP|Li and LFP|Ag@Li cells at 0.5 C. An initial capacity of 142 mAh g^−1^ and a capacity retention ratio of 92% after 300 cycles are obtained for the LFP|Ag@Li full cell at 0.5 C, while the capacity retention ratio of LFP|Li cell is nearly 71%, showing that the full cell with Ag@Li electrode is more stable during cycling. Figure 4B presents the rate performance of the full cells with Ag@Li electrode. It can be seen that the capacity of LFP|Ag@Li cell keep stable at different current density even at 10 C. The galvanostatic charge-discharge curves of LFP|Li and LFP|Ag@Li cells after 1st, 50th, and 100th cycles are shown in Figures 4C,D. The full cell with Ag@Li electrode is more stable and shows lower polarization voltage than of the full cell with bare Li electrode.

**Figure 5:**
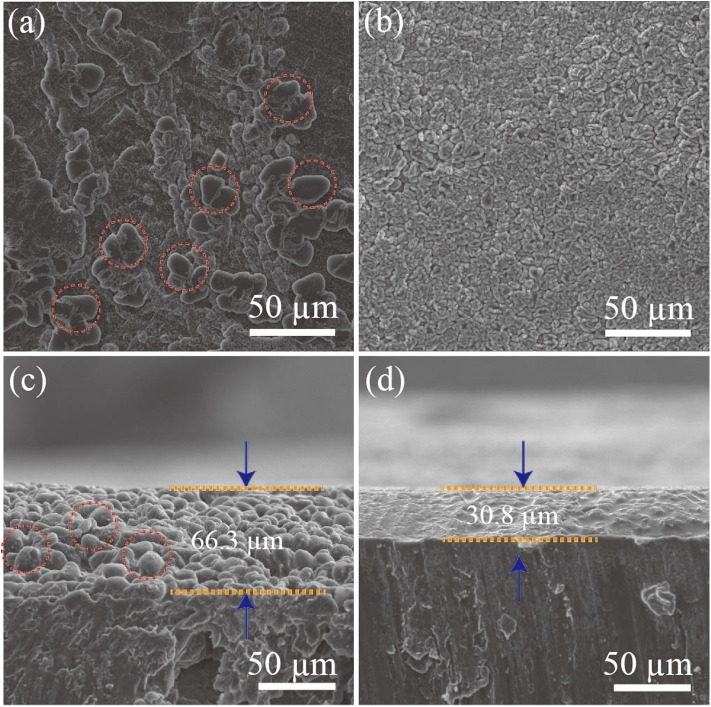
SEM images of the surface of **(a)** bare Li and **(b)** Ag@Li. Cross-sectional SEM images of **(c)** bare Li and **(d)** Ag@Li. Symmetric cells are discharged at a current density of 2.5 mA cm^−2^ with a fixed capacity of 5 mAh cm^−2^.

To further study the Li metal electrochemical deposition, a symmetric cell was discharged at a current density of 2.5 mA cm^−2^ with a fixed capacity of 5 mAh cm^−2^. As shown in Figure 5a, Li dendrites are detected on the surface of the pristine Li foil during the deposition process. It is noteworthy that Li dendrites tend to fill the interspace among the Ag particles, resulting in the lateral growth on the surface of Ag@Li electrode (Figure 5b). Moreover, obvious Li dendrites with a thickness of ~66.3 μm are detected from the cross-sectional SEM image for the bare Li electrode after 50 cycles (Figure 5c), while the Ag@Li electrode shows a dense Ag-Li alloy layer of ~30.8 μm without any Li dendrites on the electrode surface (Figure 5d). The remarkable Li dendrites inhibition ability of the Ag@Li layer is mainly caused by the strong affinity of Li with Ag, which can induce uniform deposition of Li ions during electrochemical cycling.

**Figure 6:**
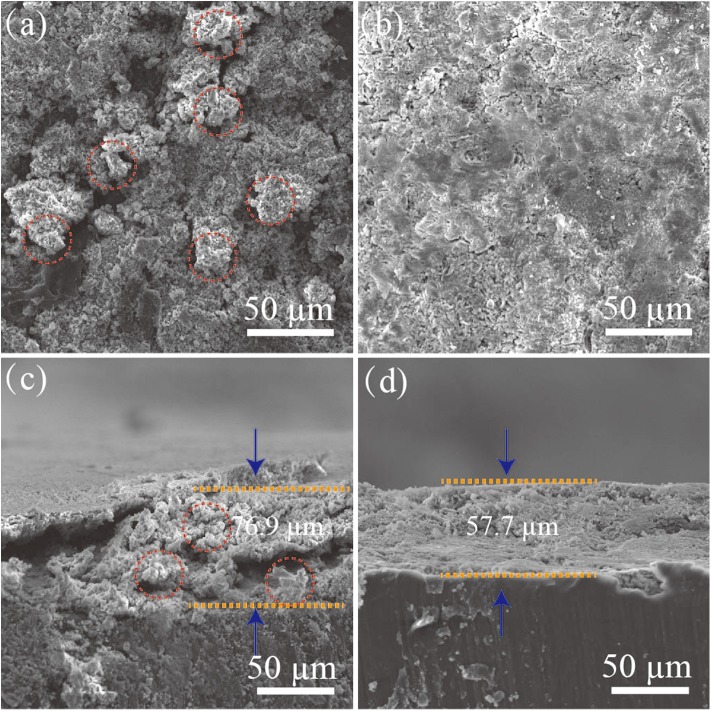
Surficial SEM images of **(a)** the bare Li and **(b)** the Ag@Li electrode. Cross-sectional SEM images of **(c)** the bare Li and **(d)** the Ag@Li electrodes in the symmetric cells after 50 cycles at a current density of 1 mA cm^−2^ with a fixed capacity of 1 mAh cm^−2^.

Figure 6 shows the SEM images of the bare Li and the Ag@Li electrodes after 50 cycles at a current density of 1 mA cm^−2^ and a fixed capacity of 1 mAh cm^−2^. The Ag@Li electrode has a flatter surface compare to that of the bare Li. From the cross-section images, cracks and dendrites emerged on the bare Li electrode surface due to the volume changes during the Li plating/string process. Oppositely, the Ag@Li electrode demonstrates a smooth boundary layer with a thickness of 57.7 μm.

Table 1 summaries the overpotential of the cells with modified Li metal anodes in recent literatures. By comparison, the overpotential of cells with Ag@Li anode in this work is 38 mV, which is competitive with other kinds of modified Li metal anode.

**Table 1:** Summary of the overpotential of the cells with modified Li metal anodes.

**Anode**	**Current density (mA cm^**−2**^)**	**Capacity (mAh cm^**−2**^)**	**Overpotential (mV)**	**References**
Li_2_S@Li	2	5	95	Chen H. et al., 2019
Li_x_Si@Li	1	1	40	Tang et al., 2018
Li_3_P@Li	0.1	0.5	60	Kim et al., 2018
Li/Cu-VAMCs	1	1	20	Wang S. H. et al., 2017
Li/VA-CuO-Cu	0.5	0.5	15	Zhang C. et al., 2018
Ag@Li	1	1	38	This work

## Conclusions

In this work, a thin lithiophilic Ag layer was coated on the bare Li surface by using thermal evaporation deposition to inhibit Li dendrites growth. The lithiophilic Ag layer provides Li nucleation sites to regulate Li ions flux for Li deposition. The excellent electrical conductivity of Ag also improves the Li ions transport capacity. The Ag@Li|Ag@Li symmetric cell shows great cycling over 85 h at current density up to 5 mA cm^−2^. After 300 cycles, a capacity retention ratio of nearly 92 % is achieved in the LFP full cell. This research gives us a new inspiration for improving the lifetime and safety of the Li metal batteries.

## Data Availability Statement

All datasets generated for this study can be found in the article.

## Author Contributions

ZZ and LZ contributed equally to this work. BT, ZZ, and LZ designed this work. ZZ synthesized the electrode and carried out the experiment. ZZ, LZ, and BT co-wrote the manuscript. All the authors gave useful suggestions, discussed the results and commented on the manuscript.

### Conflict of Interest

The authors declare that the research was conducted in the absence of any commercial or financial relationships that could be construed as a potential conflict of interest.
